# Non-negative matrix factorization algorithms generally improve topic model fits

**DOI:** 10.1007/s11222-026-10866-0

**Published:** 2026-05-05

**Authors:** Peter Carbonetto, Abhishek Sarkar, Zihao Wang, Matthew Stephens

**Affiliations:** 1 Department of Human Genetics, University of Chicago, Chicago, IL USA; 2 Vesalius Therapeutics, Cambridge, MA USA; 3 Department of Statistics, University of Chicago, Chicago, IL USA

**Keywords:** Topic models, Non-negative matrix factorization, Nonconvex optimization, Expectation maximization, Maximum-likelihood estimation

## Abstract

In an effort to develop topic modeling methods that can be quickly applied to large data sets, we revisit the problem of maximum-likelihood estimation in topic models. It is known, at least informally, that maximum-likelihood estimation in topic models is closely related to non-negative matrix factorization (NMF). Yet, to our knowledge, this relationship has not been exploited previously to fit topic models. We show that recent advances in NMF optimization methods can be leveraged to fit topic models very efficiently, often resulting in much better fits and in less time than existing algorithms for topic models. We also formally make the connection between the NMF optimization problem and maximum-likelihood estimation for the topic model, and using this result we show that the expectation maximization (EM) algorithm for the topic model is essentially the same as the classic multiplicative updates for NMF. Our methods are implemented in the R package “fastTopics”.

## Introduction

The focus of this paper is the problem of computing maximum-likelihood estimates (MLEs) of the parameters in a topic model given an $n \times m$ matrix of counts. Instead of directly computing the MLE for the topic model, we instead propose to solve a similar problem with much simpler constraints on the parameters: optimizing a non-negative matrix factorization (NMF) based on a Poisson model of the data (Lee and Seung (1999, 2001); Cichocki et al. (2011); Dhillon and Sra (2005); Févotte and Idier (2011); Hien and Gillis (2021)). Despite the fact that the topic model and Poisson NMF are well known to be closely related (Buntine (2002); Buntine and Jakulin (2006); Canny (2004); Ding et al. (2008); Faleiros et al. (2016); Gaussier and Goutte (2005); Gillis (2021); Zhou et al. (2012)), as far as we are aware this relationship has not been previously exploited for fitting topic models.

An intuition for the advantage of this approach is that the Poisson NMF optimization problem lacks the “sum-to-one” constraints, which complicate optimization. However, not all Poisson NMF algorithms exploit this benefit. Indeed, the traditional way to solve Poisson NMF—the “multiplicative updates” of Lee and Seung (2001)—is equivalent to expectation maximization (EM) (Cemgil 2009), and, as we show, is closely related to the EM algorithms traditionally used to fit topic models. (In fact, we show that these algorithms are mostly the same except for the order in which the operations are performed.) Therefore, the Poisson NMF multiplicative updates are expected to experience the same issues as EM, and this is indeed borne out by our experiments. In contrast, other recently developed algorithms for solving Poisson NMF based on co-ordinate descent (CD) (Hsieh and Dhillon 2011; Lin and Boutros 2020) do not have an existing counterpart for topic models. It has been shown that CD algorithms can greatly outperform the multiplicative updates for Poisson NMF (Hien and Gillis 2021). And here we show that CD algorithms can also be leveraged to fit topic models very efficiently, often resulting in much better fits and in less time than the existing algorithms for topic models.

A maximum-likelihood approach to topic modeling is not new, of course; one of the very first papers on topic modeling, Hofmann (1999), used a simple EM algorithm to obtain MLEs under the topic model. EM, however, can be very slow to converge to a local maximum of the likelihood (Redner and Walker (1984); Ma et al. (2000); Dwivedi et al. (2020); Kunstner et al. (2021); Zhou et al. (2011); Varadhan and Roland (2008); Henderson and Varadhan (2019)). The slow convergence of EM is sometimes viewed as a feature, not a bug: “early stopping” has been shown, both anecdotally and in theory, to result in parameter estimates that better generalize to test sets—that is, early stopping can *implicitly regularize* the MLEs (Ali et al. 2019; Gunasekar et al. 2017). We show however that this slow convergence can also sometimes cause the EM to get “stuck” in areas of the likelihood that are far away from a local maximum, resulting in very poor parameter estimates. We also show that the fast NMF algorithms can very quickly “rescue” the EM estimates, resulting in parameter estimates that are very different from and much better than the estimates produced by EM.

The maximum-likelihood approach we study in this paper contrasts with the much more widely used variational inference approach for topic models, i.e., latent Dirichlet allocation (Blei et al. (2003); Teh et al. (2007); Asuncion et al. (2009)). The benefit of variational inference is that it produces approximate posterior estimates of the model parameters, which can help to address overfitting, stabilize parameter estimates, and increase accuracy. However, the underlying computations for variational inference are more complex, making the algorithms slower and more challenging to apply to very large data sets. For these reasons, “online” variational inference algorithms have been developed (Hoffman et al. 2010; Sato 2001). But online learning algorithms bring their own challenges; for example, unlike conventional optimization algorithms, they do not guarantee that the objective will improve at each iteration, and the results of online learning are often sensitive to parameter tuning. (Markov chain Monte Carlo algorithms for posterior inference in topic models have also been used in the past e.g., Griffiths and Steyvers (2004), but MCMC is typically more computationally burdensome than variational inference.) Therefore, on balance, maximum-likelihood estimation remains an attractive option for many large data sets, especially when maximum-likelihood estimation is implemented using fast NMF algorithms, as we show here. Indeed, reframing the problem of fitting a topic model as an NMF optimization problem has already enabled us and others to efficiently fit topic models to very large single-cell data sets, in some cases with $n, m \ge \text{100,000 }$ (Chirichella et al. 2025; Dey et al. 2017; González-Blas et al. 2019; Carbonetto et al. 2023; Umans and Gilad 2025; Meir et al. 2025; Gao et al. 2024; Popp et al. 2024; Liang et al. 2024; Zhao et al. 2024; Housman et al. 2022; Hung et al. 2022; Rhodes et al. 2022; Bastide et al. 2022). The numerical experiments in this paper include two single-cell data sets, and demonstrate the benefits of applying fast NMF algorithms to fit topic models for single-cell data sets.

The algorithms for fitting topic models and Poisson NMF described in this paper are implemented in an R package, fastTopics, which is available on CRAN (https://cran.r-project.org/package=fastTopics) and on GitHub (https://github.com/stephenslab/fastTopics/).

## Poisson NMF and the multinomial topic model

In the following, we provide side-by-side descriptions of the topic model and Poisson NMF to highlight their close connection. While formal and informal connections between these two models have been made previously (Buntine (2002); Buntine and Jakulin (2006); Canny (2004); Ding et al. (2008); Faleiros et al. (2016); Gaussier and Goutte (2005); Zhou et al. (2012)). We provide a simple and more general result relating the likelihoods of the two models (Lemma 1), which we view as a more fundamental result underlying previous results.^1^

Let $\textbf{X}\in \textbf{R}_{+}^{n \times m}$ denote an $n \times m$ matrix of observed counts $x_{ij}$. For example, when analyzing text documents, *n* is the number of documents, *m* is the number of unique terms, and $x_{ij}$ is the number of times term *j* occurs in document *i*. Both Poisson NMF and the topic model can be seen as fitting different—but closely related—models of $\textbf{X}$.

The Poisson NMF model has parameters that are non-negative matrices, $\textbf{H} \in \textbf{R}_{+}^{n \times K}$ and $\textbf{W} \in \textbf{R}_{+}^{m \times K}$, where $\textbf{R}_{+}^{r \times c}$ denotes the set of non-negative, real matrices with *r* rows and *c* columns. Given a $K \ge 1$, the Poisson NMF model is

1 $$\begin{aligned} \begin{aligned} x_{ij}&\mid \textbf{H}, \textbf{W} \sim \textrm{Pois}(\lambda _{ij}) \\ \lambda _{ij}&= (\textbf{HW}^T)_{ij} = \sum _{k=1}^K h_{ik} w_{jk}, \end{aligned} \end{aligned}$$

where $h_{ij}, w_{jk}$ denote elements of matrices $\textbf{H}, \textbf{W}$, and $\textrm{Pois}(\lambda )$ denotes the Poisson distribution with rate $\lambda$. Poisson NMF can be viewed as a rank-*K* matrix factorization by noting that (1) implies $E[\textbf{X}] = \textbf{HW}^T$. So fitting a Poisson NMF essentially seeks values of $\textbf{H}$ and $\textbf{W}$ such that $\textbf{X}\approx \textbf{HW}^T$.^2^ Computing an MLE for the Poisson NMF model reduces to the following *bound-constrained* optimization problem:

2 $$\begin{aligned} \begin{array}{ll} \text{ minimize } & \ell (\textbf{X}; \textbf{H}, \textbf{W}) \\ \text{ subject } \text{ to } & \textbf{H} \ge {{\boldsymbol{0}}}, \textbf{W} \ge {{\boldsymbol{0}}}, \end{array} \end{aligned}$$

in which the objective function is

3 $$\begin{aligned} \ell (\textbf{X}; \textbf{H}, \textbf{W})~{:}{=}&\; \phi (\textbf{X}; \textbf{H}, \textbf{W}) + \Vert \textbf{H} \textbf{W}^T \Vert _{1,1} \end{aligned}$$

4 $$\begin{aligned} \phi (\textbf{X}; \textbf{H}, \textbf{W})~{:}{=}&\; -\sum _{i=1}^n \sum _{j=1}^m x_{ij} \log {{\boldsymbol{h}}}_i^T {{\boldsymbol{w}}}_j, \end{aligned}$$

where $\Vert \textbf{A}\Vert _{1,1} = \sum _{i = 1}^n \sum _{j = 1}^m |a_{ij}|$ is the $L_{1,1}$ norm of $n \times m$ matrix $\textbf{A}$, and ${{\boldsymbol{h}}}_i, {{\boldsymbol{w}}}_j$ denote, respectively, the *i*th row of $\textbf{H}$ and the *j*th row of $\textbf{W}$.

Like Poisson NMF, the multinomial topic model is also parameterized by two non-negative matrices, $\textbf{L}\in \textbf{R}_{+}^{n \times K}$, $\textbf{F}\in \textbf{R}_{+}^{m \times K}$, but the elements of these two matrices must satisfy additional “sum-to-one” constraints:

5 $$\begin{aligned} \sum _{j=1}^m f_{jk} = 1, \quad \sum _{k=1}^K l_{ik} = 1. \end{aligned}$$

Most variations of the topic model, including the aspect model (Hofmann et al. 1999), probabilistic latent semantic indexing (Hofmann 2001; Hoffman et al. 2010; Hofmann 1999) and latent Dirichlet allocation (Blei et al. 2003), are based on the same basic model: a multinomial distribution of the counts. We therefore refer to this model as the *multinomial topic model.* Given a $K \ge 2$, the multinomial topic model is

6 $$\begin{aligned} \begin{aligned} x_{i1}, \ldots , x_{im}&\mid \textbf{L}, \textbf{F} \sim \textrm{Multin}(t_i; \pi _{i1}, \ldots , \pi _{im}) \\ \pi _{ij}&= (\textbf{L}\textbf{F}^T)_{ij} = \sum _{k=1}^K l_{ik} f_{jk}, \end{aligned} \end{aligned}$$

in which $\textrm{Multin}(n; \pi _1, \ldots , \pi _m)$ is the multinomal distribution with sample size *n* and probabilities $\pi _1, \ldots , \pi _m$, and $t_i~{:}{=}\sum _{j=1}^m x_{ij}$. The multinomial topic model is also a matrix factorization because we have that $\mathbf{\Pi } = \textbf{L}\textbf{F}^T$, where $\mathbf{\Pi }$ denotes the matrix of multinomial probabilities $\pi _{ij}$ (Hofmann (1999); Singh and Gordon (2008); Steyvers and Griffiths (2007)). Computing an MLE for the multinomial topic model reduces to a *linearly constrained* optimization problem,

7 $$\begin{aligned} \begin{array}{ll} \text{ minimize } & \phi (\textbf{X}; \textbf{L}, \textbf{F}) \\ \text{ subject } \text{ to } & \textbf{L} {\boldsymbol{1}}_K = {\boldsymbol{1}}_n \\ & \textbf{F}^T {\boldsymbol{1}}_m = {\boldsymbol{1}}_K \\ & \textbf{L} \ge {\boldsymbol{0}}, \textbf{F} \ge {\boldsymbol{0}}, \end{array} \end{aligned}$$

in which ${\boldsymbol{1}}_d = (1, \ldots , 1)^T$ denotes a column vector of ones of length *d*, and $\phi$ was defined in (4).

Now we connect the Poisson non-negative matrix factorization to the multinomial topic model matrix factorization. To do so, we define a mapping between the parameter spaces for the two models (Definition 1), and then we state an equivalence between their likelihoods (Lemma 1), which leads to an equivalence in their MLEs (Corollary 1).

### Definition 1

*(Poisson NMF to multinomial topic model reparameterization)* Let $\textbf{R}_{++}^d$ denote the set of positive real vectors of length *d*, let $\textbf{R}_\textrm{row}^{r \times c}$ denote the set of $r \times c$ row-normalized matrices (non-negative matrices $\textbf{A}$ with the property that the elements in each row of $\textbf{A}$ sum to 1), and let $\textbf{R}_\textrm{col}^{r \times c}$ denote the set of $r \times c$ column-normalized matrices (non-negative matrices $\textbf{A}$ with the property that the elements in each column of $\textbf{A}$ sum to 1). For $K \ge 2$, $\textbf{H} \in \textbf{R}_{+}^{n \times K}$, $\textbf{W} \in \textbf{R}_{+}^{m \times K}$, define mapping $\textsc {PNMF-\!to-\!MTM} : \textbf{H}, \textbf{W} \mapsto \textbf{L}, \textbf{F}, {{\boldsymbol{s}}}, {{\boldsymbol{u}}}$, with $\textbf{L}\in \textbf{R}_{\textrm{row}}^{n \times K}$, $\textbf{F} \in \textbf{R}_{\textrm{col}}^{m \times K}$, ${{\boldsymbol{s}}} \in \textbf{R}_{++}^n$, ${{\boldsymbol{u}}} \in \textbf{R}_{++}^K$ by the following procedure: 
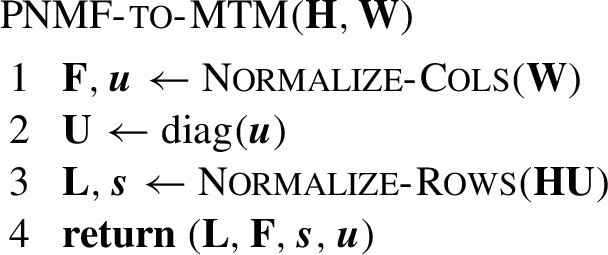
 This procedure has two subroutines, defined as follows: $\textsc {Normalize-\!Rows}(\textbf{A})$ returns a vector ${{\boldsymbol{y}}} \in \textbf{R}_{++}^r$ containing the row sums of $r \times c$ matrix $\textbf{A}$, $y_i = \sum _{j=1}^c a_{ij}$, and $\textbf{B} \in \textbf{R}_\textrm{row}^{r \times c}$, a row-normalized matrix with entries $b_{ij} = a_{ij} / y_i$; and $\textsc {Normalize-\!Cols}(\textbf{A})$ returns a vector ${{\boldsymbol{y}}} \in \textbf{R}_{++}^c$ containing the column sums of $\textbf{A}$, $y_j = \sum _{i=1}^r a_{ij}$, and $\textbf{B} \in \textbf{R}_\textrm{col}^{r \times c}$, a column-normalized matrix with entries $b_{ij} = a_{ij} / y_j$. We also define $\textrm{diag}({{\boldsymbol{a}}})$ as the $n \times n$ diagonal matrix $\textbf{A}$ with diagonal entries given by the elements of vector ${{\boldsymbol{a}}}$.

$\textsc {Normalize-\!Rows}$ also defines a mapping $\textsc {Normalize-\!Rows} : \textbf{A} \mapsto \textbf{B}, {{\boldsymbol{y}}}$, with $\textbf{A} \in \textbf{R}_{+}^{r \times c}$, $\textbf{B} \in \textbf{R}_{\textrm{row}}^{r \times c}$, $\textbf{y} \in \textbf{R}_{++}^r$. If each row of $\textbf{A}$ has at least one positive element, then this mapping is one-to-one, and therefore $\textsc {Normalize-\!Rows}$ defines a *change of variables* from non-negative matrices $\textbf{A}$ to row-normalized matrices $\textbf{B}$ and positive vectors ${{\boldsymbol{y}}}$. Similarly, $\textsc {Normalize-\!Cols} : \textbf{A} \mapsto \textbf{B}, {{\boldsymbol{y}}}$ defines a change of variables from non-negative matrices $\textbf{A}$ to column-normalized matrices $\textbf{B}$ and positive vectors ${{\boldsymbol{y}}}$ (provided that each column of $\textbf{A}$ has at least one positive element). These together imply that $\textsc {MTM-\!to-\!PNMF}$ defines a change of variables from non-negative matrices $\textbf{H}, \textbf{W}$ to positive vectors ${{\boldsymbol{s}}}, {{\boldsymbol{u}}}$, row-normalized matrices $\textbf{L}$, and column-normalized matrices $\textbf{F}$. The $\textbf{F}$ and $\textbf{L}$ satisfy the sum-to-one constraints (5). The inverse mapping, $\textsc {MTM-\!to-\!PNMF}~{:}{=}~ \textsc {PNMF-\!to-\!MTM}^{-1} : \textbf{L}, \textbf{F}, {{\boldsymbol{s}}}, {{\boldsymbol{u}}} \mapsto \textbf{H}, \textbf{W}$, is $\textbf{W} \leftarrow \textbf{F}\textbf{U}$, $\textbf{H} \leftarrow \textbf{S} \textbf{L}\textbf{U}^{-1}$, where $\textbf{U}~{:}{=}~ \textrm{diag}({{\boldsymbol{u}}})$, $\textbf{S}~{:}{=}~ \textrm{diag}({{\boldsymbol{s}}})$.

### Lemma 1

(Equivalence of Poisson NMF and multinomial topic model likelihoods) Denote the Poisson NMF likelihood by $p_{\textrm{PNMF}}({\textbf{X}} \mid \textbf{H}, \textbf{W})$ and denote the multinomial topic model likelihood by $p_{\textrm{MTM}}({\textbf{X}} \mid \textbf{L}, \textbf{F})$. Assume $\textbf{H} \in \textbf{R}_{+}^{n \times K}$ and $\textbf{W} \in \textbf{R}_{+}^{m \times K}$, define $t_i~{:}{=}~ \sum _{j=1}^m x_{ij}$, and let $\textbf{L}, \textbf{F}, {{\boldsymbol{s}}}, {{\boldsymbol{u}}}$ be the result of applying $\textsc {PNMF-\!to-\!MTM}$ to $\textbf{H}, \textbf{W}$. Then we have that

8 $$\begin{aligned} p_{\textrm{PNMF}}({\textbf{X}} \mid \textbf{H}, \textbf{W}) = p_{\textrm{MTM}}({\textbf{X}} \mid \textbf{L}, \textbf{F}) \prod _{i=1}^n \textrm{Pois}(t_i; s_i), \end{aligned}$$

where $\textrm{Pois}(x; \lambda )$ denotes the probability mass function of the Poisson distribution at *x* with rate $\lambda$.

### Proof

The result is obtained by applying the following identity relating the multinomial and Poisson distributions (Fisher 1922; Good 1986):

9 $$\begin{aligned} \prod _{j=1}^m \textrm{Pois}(x_j; \lambda _j) = \textrm{Multin}({\boldsymbol{x}}; t, \lambda _1/s, \ldots , \lambda _m/s) \textrm{Pois}(t; s), \end{aligned}$$

where ${\boldsymbol{x}} = (x_1, \dots , x_m)$, $\lambda _1, \ldots , \lambda _m \in \textbf{R}_{+}$, $s~{:}{=}~ \sum _{j=1}^m \lambda _j$, $t~{:}{=}~ \sum _{j=1}^m x_j$, and $\textrm{Multin}({{\boldsymbol{x}}}; n, \pi _1, \ldots , \pi _m)$ denotes the probability mass function of the multinomial distribution at ${{\boldsymbol{x}}} = (x_1, \ldots , x_m)$ with sample size *n* and probabilities $\pi _1, \ldots , \pi _m$. $\square$

Now we use this lemma to justify solving the Poisson NMF optimization problem in order to achieve maximum-likelihood estimation in the multinomial topic model. First, consider an *augmented form* of the multinomial topic model optimization problem:

10 $$\begin{aligned} \begin{array}{ll} \text{ minimize } & \phi _{\textrm{aug}}(\textbf{X}; \textbf{L}, \textbf{F}, {{\boldsymbol{s}}}) \\ \text{ subject } \text{ to } & \textbf{L} {\boldsymbol{1}}_K = {\boldsymbol{1}}_n \\ & \textbf{F}^T {\boldsymbol{1}}_m = {\boldsymbol{1}}_K \\ & \textbf{L} \ge {\boldsymbol{0}}, \textbf{F} \ge {\boldsymbol{0}}, {{\boldsymbol{s}}} \ge {\boldsymbol{0}}, \end{array} \end{aligned}$$

in which the augmented objective is

11 $$\begin{aligned} \phi _{\textrm{aug}}(\textbf{X}; \textbf{L}, \textbf{F}, {{\boldsymbol{s}}})~{:}{=}~ \phi (\textbf{X}; \textbf{L}, \textbf{F}) + \psi (\textbf{X}; {{\boldsymbol{s}}}) \end{aligned}$$

12 $$\begin{aligned} \psi (\textbf{X}; {{\boldsymbol{s}}})~{:}{=}~ \sum _{i=1}^n s_i - \sum _{i=1}^n \sum _{j=1}^m x_{ij} \log s_i. \end{aligned}$$

Notice that solutions to (10) are also solutions to (7) because the objective and constraints for the $\textbf{L}$ and $\textbf{F}$ have not changed. The Poisson NMF objective $\ell (\textbf{X}; \textbf{H}, \textbf{W})$ is equal to the Poisson log-likelihood, $\log p_{\textrm{PNMF}}({\textbf{X}} \mid \textbf{H}, \textbf{W})$ (ignoring constant terms), and the multinomial topic model augmented objective $\phi _{\textrm{aug}}(\textbf{X}; \textbf{L}, \textbf{F}, \textbf{s})$ is equal to the logarithm of the right-hand side of (8) (again, ignoring constant terms). This means that any optimization algorithm that improves the Poisson NMF objective $\ell (\textbf{X}; \textbf{H}, \textbf{W})$ will also improve the augmented objective $\phi _{\textrm{aug}}(\textbf{X}; \textbf{L}, \textbf{F}, \textbf{s})$ so long as $\textsc {PNMF-\!to-\!MTM}$ is used to to recover $\textbf{L}, \textbf{F}, {{\boldsymbol{s}}}$ from $\textbf{H}, \textbf{W}$. We formalize the relationship between the two optimization problems in the following corollary.

### Corollary 1

(Relationship between MLEs for Poisson NMF and multinomial topic model) Let $\hat{\textbf{H}} \in \textbf{R}_{+}^{n \times K}, \hat{\textbf{W}} \in \textbf{R}_{+}^{m \times K}$ denote MLEs for the Poisson NMF model,^3^

13 $$\begin{aligned} \hat{\textbf{H}}, \hat{\textbf{W}} \in \underset{\textbf{H} \in \textbf{R}_{+}^{n \times K}, \textbf{W} \in \textbf{R}_{+}^{m \times K}}{\textrm{argmax}} p_{\textrm{PNMF}}(\textbf{X}\mid \textbf{H}, \textbf{W}). \end{aligned}$$

Equivalently, $\hat{\textbf{H}}, \hat{\textbf{W}}$ can be defined as a solution to (2). If $\hat{\textbf{L}}, \hat{\textbf{F}}$ are obtained by applying $\textsc {PNMF-\!to-\!MTM}$ to $\hat{\textbf{H}}, \hat{\textbf{W}}$, then these are also MLEs for the multinomial topic model,

14 $$\begin{aligned} \hat{\textbf{L}}, \hat{\textbf{F}} \in \underset{\textbf{L} \,\in \, \textbf{R}_{\textrm{row}}^{n \times K}, \, \textbf{F} \,\in \, \textbf{R}_{\textrm{col}}^{m \times K}}{\textrm{argmax}} \; p_{\textrm{MTM}}(\textbf{X}\mid \textbf{L}, \textbf{F}). \end{aligned}$$

Equivalently, $\hat{\textbf{L}}, \hat{\textbf{F}}$ are a solution to (7).

Conversely, let $\hat{\textbf{L}} \in \textbf{R}_{\textrm{row}}^{n \times K}, \hat{\textbf{F}} \in \textbf{R}_{\textrm{col}}^{m \times K}$ denote multinomial topic model MLEs, set $\hat{{\boldsymbol{s}}} = {{\boldsymbol{t}}}~{:}{=}~ (t_1, \ldots , t_n)$, and choose any $\hat{{\boldsymbol{u}}} \in \textbf{R}_{++}^K$. If $\hat{\textbf{H}}, \hat{\textbf{W}}$ are obtained by applying $\textsc {MTM-\!to-\!PNMF}$ to $\hat{\textbf{L}}, \hat{\textbf{F}}, \hat{{\boldsymbol{s}}}, \hat{{\boldsymbol{u}}}$, these are also Poisson NMF MLEs (13).

### Proof

We prove this result using “equivalent optimization problems” (Boyd and Vandenberghe 2004). Since $\textsc {PNMF-\!to-\!MTM}$ defines a change of variables, we can apply the change of variables to (10) to obtain an equivalent optimization problem with optimization variables $\textbf{H}, \textbf{W}$:

15 $$\begin{aligned} \begin{array}{ll} \text{ minimize } & \phi _{\textrm{aug}}(\textbf{X}; \textsc {PNMF-\!to-\!MTM}(\textbf{H}, \textbf{W})) \\ \text{ subject } \text{ to } & \textbf{H} \ge {{\boldsymbol{0}}}, \textbf{W} \ge {{\boldsymbol{0}}}, \end{array} \end{aligned}$$

in which the ${{\boldsymbol{u}}}$ returned by PNMF-to-MTM is ignored. From Lemma 1, we can rewrite (15) as

16 $$\begin{aligned} \begin{array}{ll} \text{ minimize } & \ell (\textbf{X}; \textbf{H}, \textbf{W}) + \text{ const } \\ \text{ subject } \text{ to } & \textbf{H} \ge {{\boldsymbol{0}}}, \textbf{W} \ge {{\boldsymbol{0}}}, \end{array} \end{aligned}$$

which is exactly the Poisson NMF optimization problem (ignoring terms that do not depend on $\textbf{H}$ or $\textbf{W}$). Therefore, the augmented optimization problem (10) and the Poisson NMF optimization problem (2) are related to each other by the change of variables $\textsc {PNMF-\!to-\!MTM}(\textbf{H}, \textbf{W}) = (\textbf{L}, \textbf{F}, {{\boldsymbol{s}}}, {{\boldsymbol{u}}})$. Since solutions to the augmented optimization problem (10) are also solutions to the original problem (7), it follows that Poisson NMF MLEs $\hat{\textbf{H}}, \hat{\textbf{W}}$ recover multinomial topic model MLEs $\hat{\textbf{L}}, \hat{\textbf{F}}$. The reverse—that multinomial topic model MLEs $\hat{\textbf{L}}, \hat{\textbf{F}}$ recover Poisson NMF MLEs $\hat{\textbf{H}}, \hat{\textbf{W}}$—requires the additional step of solving $\hat{\textbf{s}}~{:}{=}~ \textrm{argmin}_{{{\boldsymbol{s}}} \,\in \, \textbf{R}_{++}^n} \psi ({{\boldsymbol{s}}}) = \textrm{argmax}_{{{\boldsymbol{s}}} \,\in \, \textbf{R}_{++}^n} \prod _{i=1}^n \textrm{Pois}(t_i; s_i)$, which has unique solution $\hat{{\boldsymbol{s}}} = {{\boldsymbol{t}}}$ provided that $t_1, \ldots , t_n > 0$. $\square$

### Remark 1

Since $\textbf{H}, \textbf{W}$ are not uniquely identifiable—consider that multiplying the *k*th column of $\textbf{H}$ by $a_k \ne 0$ and dividing the *k*th column of $\textbf{W}$ by $a_k$ does not change $\textbf{H} \textbf{W}^T$—one way to avoid this non-identifiability is to impose constraints or penalty terms to the objective. However, introducing constraints or penalties on $\textbf{H}, \textbf{W}$ (or $\textbf{L}, \textbf{F}$) may break the above equivalence. We note one form of penalized objective that preserves the equivalence:

17 $$\begin{aligned} \phi ^{\star }(\textbf{X}; \textbf{L}, \textbf{F})~{:}{=}~ \phi (\textbf{X}; \textbf{L}, \textbf{F}) + \rho ^{\textrm{MTM}}(\textbf{F}), \end{aligned}$$

where

18 $$\begin{aligned} \rho ^{\textrm{MTM}}(\textbf{F})~{:}{=}~ -\sum _{j=1}^m \sum _{k=1}^K (a_{jk} - 1) \log f_{jk}, \end{aligned}$$

and $a_{jk} > 1$, $j = 1, \ldots , m$, $k = 1, \ldots , K$. The equivalent penalized objective for Poisson NMF is

19 $$\begin{aligned} \ell ^{\star }(\textbf{X}; \textbf{H}, \textbf{W})~{:}{=}~ \ell (\textbf{X}; \textbf{H}, \textbf{W}) + \rho ^{\textrm{PNMF}}(\textbf{W}), \end{aligned}$$

where

20 $$\begin{aligned} \rho ^{\textrm{PNMF}}(\textbf{W})~{:}{=}~\;&- \sum _{j=1}^m \sum _{k=1}^K (a_{jk} - 1) \log w_{jk} \nonumber \\&+ \sum _{j=1}^m \sum _{k=1}^K b_k w_{jk}, \end{aligned}$$

and $b_k > 0$, $k = 1, \ldots , K$. The $a_{jk}, b_k$ control the shape and strength of these penalties. (Setting $a_{jk} = 1, b_k = 0$ recovers the unpenalized objectives.) Minimizing $\phi ^{\star }(\textbf{X}; \textbf{L}, \textbf{F})$ corresponds to MAP estimation of $\textbf{L}, \textbf{F}$ with Dirichlet priors on $\textbf{F}$ and uniform priors on $\textbf{L}$ (Sontag et al. 2011), and minimizing $\ell ^{\star }(\textbf{X}; \textbf{H}, \textbf{W})$ corresponds to MAP estimation of $\textbf{W}, \textbf{H}$ with gamma priors on $\textbf{W}$ and uniform priors on $\textbf{H}$ (Canny 2004; Cemgil 2009; Ma et al. 2011). The equivalence of MAP estimation with these specific priors is a slight generalization of Corollary 1 (see Appendix A).

Lemma 1 and Corollary 1 are more general than previous results (Buntine and Jakulin 2006; Ding et al. 2008; Gaussier and Goutte 2005; Gillis 2021) because they apply to *any* $\textbf{H}, \textbf{W}$ and $\textbf{L}, \textbf{F}$ from Definition 1, not only a fixed point of the likelihood or objective. See Buntine (2002); Faleiros et al. (2016); Zhou et al. (2012) for other related results.

In short, Lemma 1 tells us that Poisson NMF and the multinomial topic model are Poisson and multinomial formulations of the same matrix factorization method. In particular, their shared ability to recover a decomposition into “parts” or “topics” is suggested by these formal connections.

Although Poisson NMF and the multinomial topic model achieve similar ends, the two methods still possess different advantages: Poisson NMF has an advantage in computation because it avoids the sum-to-one constraints, whereas the multinomial topic model has the advantage in interpretation because the parameters $f_{jk}, l_{ik}$ can be compared across topics *k* (the Poisson NMF parameters $h_{ik}, w_{jk}$ cannot due to the undetermined column-scaling ${{\boldsymbol{u}}}$). Therefore, by switching between the two models, we can have the advantages of both.

## Poisson NMF algorithms

Corollary 1 implies that *any algorithm for maximum-likelihood estimation in Poisson NMF is also an algorithm for maximum-likelihood estimation in the multinomial topic model.* (This also means that the NP-hardness (Arora et al. 2012; Vavasis 2010) of the two problems is related.) Fitting the Poisson NMF model involves solving (2), which we restate here in a slightly different way:

21 $$\begin{aligned} \begin{array}{ll} \text{ minimize } & \displaystyle \ell (\textbf{X}; \textbf{H},\textbf{W}) = \sum _{i=1}^n \sum _{j=1}^m {{\boldsymbol{h}}}_i^T {{\boldsymbol{w}}}_j - x_{ij} \log ({{\boldsymbol{h}}}_i^T {{\boldsymbol{w}}}_j) \\ \text{ subject } \text{ to } & \textbf{H} \ge \textbf{0}, \textbf{W} \ge \textbf{0}, \end{array} \end{aligned}$$

Here, ${{\boldsymbol{h}}}_i$ and ${{\boldsymbol{w}}}_j$ denote column vectors containing, respectively, the *i*th row of $\textbf{H}$ and the *j*th row of $\textbf{W}$, and we assume $K \ge 2$.

To facilitate comparisons of different algorithms for solving (21), in the next section we introduce an “Alternating Poisson Regression” framework for solving (21), then we describe the algorithms we have implemented, drawing on recent work and our own experimentation. See also Hien and Gillis (2021) for a detailed comparison of Poisson NMF algorithms.

### Alternating Poisson Regression for Poisson NMF

Alternating Poisson Regression arises from solving (21) by switching between optimizing over $\textbf{H}$ with $\textbf{W}$ fixed, and optimizing over $\textbf{W}$ with $\textbf{H}$ fixed. This is an example of a block-coordinate descent algorithm (Wright (2015); Bertsekas (1999)) where the two “blocks” are $\textbf{H}$ and $\textbf{W}$. It is analogous to “alternating least squares” for matrix factorization with Gaussian errors.

We point out two simple but important facts. First, by symmetry of (21), optimizing $\textbf{H}$ given $\textbf{W}$ has the same form as optimizing $\textbf{W}$ given $\textbf{H}$. Second, because of the separability of the sum in (21), optimizing $\textbf{W}$ given $\textbf{H}$ splits into *m* independent *K*-dimensional subproblems of the following form:

22 $$\begin{aligned} \begin{array}{l@{\;}l} \text{ minimize } & \ell _j({{\boldsymbol{w}}}_j)~{:}{=}~ \sum _{i=1}^n {{\boldsymbol{h}}}_i^T{{\boldsymbol{w}}}_j - x_{ij} \log ({{\boldsymbol{h}}}_i^T{{\boldsymbol{w}}}_j) \\ \text{ subject } \mathrm{ to } & {{\boldsymbol{w}}}_j \ge {{\boldsymbol{0}}}, \end{array} \end{aligned}$$

for $j = 1, \dots , m$. (And similarly for optimizing $\textbf{H}$ given $\textbf{W}$.) Because the *m* subproblems (22) are independent, their solutions can be pursued in parallel. While both of these observations are simple, neither of them hold for the multinomial topic model due to the sum-to-one constraints.

Subproblem (22) is itself a well-studied maximum-likelihood estimation problem (McLachlan and Krishnan (2008); Lange and Carson (1984); Lucy (1974); Molina et al. (2001); Richardson (1972); Shepp and Vardi (1982); Vardi et al. (1985)), and it is equivalent to computing an MLE of ${{\boldsymbol{b}}}~{:}{=}~ (b_1, \ldots , b_K)^T \ge {{\boldsymbol{0}}}$ in an additive Poisson regression model:

23 $$\begin{aligned} \begin{aligned} y_i&\sim \textrm{Pois}(\mu _i) \\ \mu _i&= \textstyle \sum _{k=1}^K a_{ik} b_k, \end{aligned} \end{aligned}$$

in which ${{\boldsymbol{y}}} = (y_1, \ldots , y_n)^T \in \textbf{R}_{+}^n$ and $\textbf{A} \in \textbf{R}_{+}^{n \times K}$. Consider a function that returns an MLE of ${{\boldsymbol{b}}}$ for (23),

24 $$\begin{aligned} \textsc {Fit-\!Pois-\!Reg}(\textbf{A}, {{\boldsymbol{y}}})~{:}{=}~ \underset{{{\boldsymbol{b}}} \, \in \, \textbf{R}_{+}^K}{\textrm{argmax}} \; p_{\textrm{PR}}({{\boldsymbol{y}}} \mid \textbf{A}, {{\boldsymbol{b}}}), \end{aligned}$$

where $p_{\textrm{PR}}({{\boldsymbol{y}}} \mid \textbf{A}, {{\boldsymbol{b}}})$ denotes the likelihood under the Poisson regression model (23). Any algorithm that solves (24) be can applied iteratively to solve the Poisson NMF problem (21). This idea, which we call “alternating Poisson regression for Poisson NMF”, is formalized in Algorithm 1.

**Algorithm 1:**
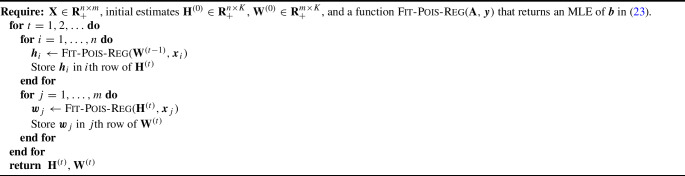
Alternating Poisson Regression for Poisson NMF. Here, ${{\boldsymbol{x}}}_i$ denotes a row of ${\textbf{X}}$ and ${{\boldsymbol{x}}}_j$ denotes a column of $\textbf{X}$.

### Specific algorithms

We now consider different approaches to solving $\textsc {Fit-\!Pois-\!Reg}(\textbf{A}, {{\boldsymbol{y}}})$, which, when inserted into Algorithm 1, produce different Poisson NMF algorithms. These algorithms are closely connected to existing algorithms for Poisson NMF and/or the multinomial topic model (Table 1).

#### Expectation maximization

There is a long history of solving the Poisson regression problem (24) by EM (Dempster et al. (1977); De Pierro (1993); McLachlan and Krishnan (2008); Krishnan (1995); Lange and Carson (1984); Lucy (1974); Meng and Van Dyk (1997); Molina et al. (2001); Richardson (1972); Shepp and Vardi (1982); Vardi et al. (1985); Vardi and Lee (1993)). The EM updates for this problem consist of iterating the following updates:

25 $$\begin{aligned} \bar{z}_{ik}&= y_i a_{ik} b_k / \mu _i \end{aligned}$$

26 $$\begin{aligned} b_k&= \frac{\sum _{i=1}^n \bar{z}_{ik}}{\sum _{i=1}^n a_{ik}}, \end{aligned}$$

where $\bar{z}_{ik}$ represents a posterior expectation in an equivalent augmented model (see Appendix A).

This EM algorithm is closely connected to the multiplicative update rules for Poisson NMF (Lee and Seung 2001): combining the E step (25) and M step (26) with the substitutions used in Algorithm 1 yields

27 $$\begin{aligned} h_{ik}^{\textrm{new}}&\leftarrow h_{ik} \times \frac{\sum _{j=1}^m x_{ij} w_{jk} / \lambda _{ij}}{\sum _{j=1}^m w_{jk}} \end{aligned}$$

28 $$\begin{aligned} w_{jk}^{\textrm{new}}&\leftarrow w_{jk} \times \frac{\sum _{i=1}^n x_{ij} h_{ik} / \lambda _{ij}}{\sum _{i=1}^n h_{ik}}, \end{aligned}$$

which are precisely the multiplicative updates for Poisson NMF. See Appendix A for the derivation. Additionally, applying $\textsc {PNMF-\!to-\!MTM}$ to the multiplicative updates (27, 28) recovers the EM updates for the multinomial topic model (Asuncion et al. (2009); Buntine (2002); Gaussier and Goutte (2005); Hofmann (2001)). See Appendix A for the derivation. Therefore, when $\textsc {Fit-\!Pois-\!Reg}(\textbf{A}, {{\boldsymbol{y}}})$ is solved using EM, Algorithm 1 can be viewed as implementing “stepwise” variants of the multiplicative updates for Poisson NMF, or EM for the multinomial topic model (Table 1). By “stepwise”, we mean that the update order suggested by Algorithm 1 is to iterate the E and M steps for the first row of $\textbf{H}$, then for the second row of $\textbf{H}$, and so on, followed by updates to rows of $\textbf{W}$. This is in contrast to a typical EM algorithm in which all E-step updates are performed first, then all M-step updates are performed.

**Table 1:** Relationship between maximum-likelihood estimation algorithms for the additive Poisson regression model, Poisson NMF, and the multinomial topic model. Abbreviations used: EM = expectation maximization (Shepp and Vardi 1982; Hofmann 1999), MU = multiplicative updates (Lee and Seung 1999, 2001), CD = co-ordinate descent (Bouman and Sauer 1996), CCD = cyclic co-ordinate descent (Hsieh and Dhillon 2011), SCD = sequential co-ordinate descent (Lin and Boutros 2020).

Additive Poisson regression	Poisson NMF	topic model
EM	MU	EM
CD	CCD, SCD	*none*

#### Co-ordinate descent

Co-ordinate descent is an alternative to EM that iteratively optimizes a single co-ordinate while the remaining co-ordinates are fixed (see also (Bouman and Sauer 1996)). For the Poisson regression problem, each 1-d optimization is straightforward to implement via Newton’s method,

29 $$\begin{aligned} b_k^{\textrm{new}} \leftarrow \max \{0, b_k - \alpha _k g_k/q_k \}, \end{aligned}$$

where $\alpha _k \ge 0$ is a step size that can be determined by a line search or some other method, and $g_k$ and $q_k$ are the partial derivatives with respect to the negative log-likelihood $\ell _{\textrm{PR}}({{\boldsymbol{b}}})~{:}{=}~ -\log p_{\textrm{PR}}({{\boldsymbol{y}}} \mid \textbf{A}, {{\boldsymbol{b}}})$,

30 $$\begin{aligned} g_k&~{:}{=}~~\frac{\partial \ell _{\textrm{PR}}}{\partial b_k} = \sum _{i=1}^n a_{ik} \left( 1 - \frac{y_i}{\mu _i}\right) \end{aligned}$$

31 $$\begin{aligned} q_k&~{:}{=}~~\frac{\partial ^2 \ell _{\textrm{PR}}}{\partial b_k^2} = \sum _{i=1}^n \frac{y_i a_{ik}^2}{\mu _i^2}. \end{aligned}$$

Several Poisson NMF algorithms, including cyclic co-ordinate descent (CCD) (Hsieh and Dhillon 2011), sequential co-ordinate descent (SCD) (Lin and Boutros 2020) and scalar Newton (SN) (Hien and Gillis 2021), can be viewed as implementing variants of this CD approach. That is, these approaches are essentially Algorithm 1 in which $\textsc {Fit-\!Pois-\!Reg}(\textbf{A}, {{\boldsymbol{y}}})$ is solved by CD. The CCD and SCD methods appear to be independent developments of the same or very similar algorithm; they both take a full (feasible) Newton step, setting $\alpha _k = 1$ when $b_k - \alpha _k g_k/q_k > 0$. By foregoing a line search to determine $\alpha _k$, the update is not guaranteed to decrease the objective $\ell _{\textrm{PR}}({{\boldsymbol{b}}})$. The SN method was developed to remedy this issue, with a step size scheme that always produces a decrease while avoiding the expense of a line search. However, Hien and Gillis (2021) compared SN with CCD and found that CCD usually performed best in real data sets despite not having a line search.

Although the CD approach is straightforward for Poisson NMF, it is not straightforward for the multinomial topic model due to the sum-to-one constraints.

## Numerical experiments

To summarize, we have described two variants of Algorithm 1 for Poisson NMF (Table 1): the first fits an “additive Poisson regression” model using EM, and is essentially the same as existing EM algorithms for Poisson NMF and the multinomial topic model (including the Poisson NMF multiplicative updates); the second uses co-ordinate descent (CD) to fit the additive Poisson regression model, and has no equivalent among existing algorithms for the multinomial topic model. In the remainder, we refer to these two variants of Algorithm 1 as “EM” and “CD”.

**Fig. 1:**
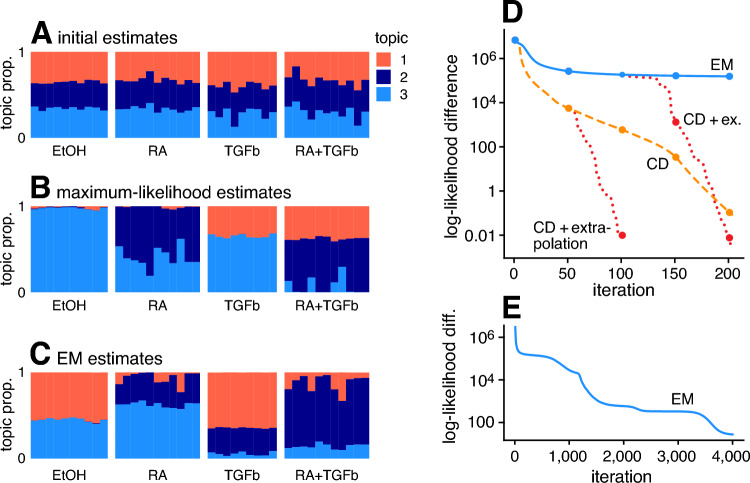
Results of fitting multinomial topic models to the MCF-7 data set (Sanford et al. 2020) with $K = 3$. Plots A–C show estimates of the $41 \times 3$ matrix $\textbf{L}$: the initial estimates (obtained by running 4 EM updates); the MLE (obtained by running many CD updates, starting from the initial estimates); and the estimates obtained by running 200 EM updates starting from the initial estimates. Each estimate of $\textbf{L}$ is visualized using a “Structure plot” (Rosenberg 2002), which is a stacked bar chart in which the bar heights are given by the elements of $\textbf{L}$. Plots D, E show the improvement in the multinomial topic model fits over time. Multinomial topic model log-likelihoods are shown relative to the log-likelihood of the multinomial topic model at the MLE (B); points highest on the y-axis indicate the worst log-likelihoods.

We begin with an in-depth example on a real data set to illustrate the differences between the EM and CD algorithms. The data for this example are RNA-sequencing read counts for $n = 41$ samples and $m = \text{1 }6, 773$ genes from Sanford et al. (2020). This data set provides a ground truth of sorts for fitting the topic model: the data are gene expression measurements taken after human MCF-7 cells were exposed to either ethanol (EtOH), retinoic acid (RA), TGF-$\beta$, or the combination of RA and TGF-$\beta$. Therefore, the topic model with $K = 3$ topics should reflect the three exposures—EtOH, RA and TGF-$\beta$—and samples in the combined exposure should be modeled as a combination of the RA and TGF-$\beta$ topics. Indeed, the MLE we obtained by running the Poisson NMF algorithm for a long time (with CD updates) largely produced the expected result: the samples in the ethanol condition were mostly represented by a single topic (the “ethanol topic”); the samples in the combination treatment were an even combination of the RA and TGF-$\beta$ topics; and the samples exposed to either RA and TGF-$\beta$ were represented as combinations of the ethanol and RA topics or the ethanol and TGF-$\beta$ topics (Fig. 1B). The steps taken to prepare these data for topic modeling are detailed in Appendix C. The code implementing this experiment is provided in a Zenodo repository (Carbonetto et al. 2024), and is available online at https://github.com/stephenslab/fastTopics-experiments/.

To compare the performance of EM and CD on this data set, we first initialized the Poisson NMF parameters at random, then we ran 4 EM updates to slightly improve upon this random initialization. The resulting initial estimate of $\textbf{L}$ is shown in Fig. 1A. Next, starting from this initial estimate, we ran 200 EM updates or 200 CD updates. (“Update” here means one iteration of the outer loop of Algorithm 1.) The CD updates produced estimates very close to the MLE; the distance to the MLE in log-likelihood units was just 0.079 (Fig. 1D). By contrast, the EM estimates remained very far away from the MLE after 200 iterations, at a distance of over 150,000 log-likelihood units (Fig. 1D). The EM estimates after 200 iterations (Fig. 1C) were also *qualitatively* very different from the MLE. The EM estimates are arguably less interpretable because they do not correspond as well to the exposures.

To rule out the possibility the EM was unlucky and had settled into a different local maximum of the likelihood, we ran many more EM updates. Eventually, EM recovered the same MLE (Fig. 1E). Therefore, the very slow progress of EM could not be explained by having converged to a less optimal stationary point. One could conclude from this comparison that good estimates could be obtained simply by running the EM updates for a long time. However, this is often impractical for larger data sets.

Another algorithmic innovation we present here is the use of the extrapolation method (Ang and Gillis 2019) to accelerate convergence of the Poisson NMF algorithm. The idea behind the extrapolation method, which builds on the method of parallel tangents (Luenberger and Ye 2015), is to avoid the “zigzagging” behaviour of the block-coordinate updates by iteratively adapting the step size according to the performance of the extrapolated updates compared to the non-extrapolated updates. The additional operations needed to implement the extrapolated updates impose minimal overhead. The extrapolation method was originally applied to Frobenius-norm NMF, and to our knowledge it has not been used to accelerate algorithms for Poisson NMF, or for fitting topic models. (More details on the extrapolation method and its implementation for Poisson NMF are given in Appendix B.) To illustrate the benefits of extrapolation, we activated the extrapolation method at iteration 50 of the CD algorithm. Doing so allowed the CD updates to recover the MLE much more quickly than the non-extrapolated CD updates (Fig. 1D). Further, when we applied the extrapolated CD updates to the EM estimates, they quickly “rescued” the poor EM estimates (Fig. 1D), further suggesting that the slow progress of EM was not due to some fundamental difficulty of the objective, but rather due to properties of the EM updates.

In summary, the results from this example suggest the potential for NMF methods—in particular, Algorithm 1 with CD updates plus extrapolation—to improve maximum-likelihood estimation for the multinomial topic model. To assess this more systematically, we performed comparisons of the EM and CD variants in a variety of data sets (Table 2): two text data sets (Globerson et al. 2007; Rennie 2007) that have been used to evaluate topic modeling methods (e.g., Asuncion et al. 2009; Wallach 2006); and two single-cell RNA sequencing (scRNA-seq) data sets (Montoro et al. 2018; Zheng et al. 2017). Appendix C gives additional details on these data sets and the experiment setup. All the algorithms compared in these experiments were implemented in the fastTopics R package. These R implementations include the enhancements described in Appendix B intended to make the algorithms more efficient and numerically stable. The code implementing these experiments is provided in a Zenodo repository (Carbonetto et al. 2024), and is available online at https://github.com/stephenslab/fastTopics-experiments/.

**Table 2:** Data sets used in the experiments.

name	rows	columns	nonzeros
NeurIPS	2,483	14,036	3.7%
newsgroups	18,774	55,911	0.2%
epithelial airway	7,193	18,388	9.3%
68k PBMC	68,579	20,387	2.7%

To reduce the possibility that multiple optimizations converge to different local maxima of the likelihood, which could complicate the comparisons, we first ran 1,000 EM updates—that is, 1,000 iterations of the outer loop of Algorithm 1—then we examined the performance of the algorithms *after* this initialization phase. Therefore, in our comparisons we assessed the extent to which the different algorithms improved upon this initialization. Another issue was that it was not always practical to run the optimization algorithms long enough to obtain an accurate MLE. Therefore, instead of comparing the estimates to the MLE, like we did in the example above, we used as a reference point the best estimate (in log-likelihood) that was obtained.

**Fig. 2:**
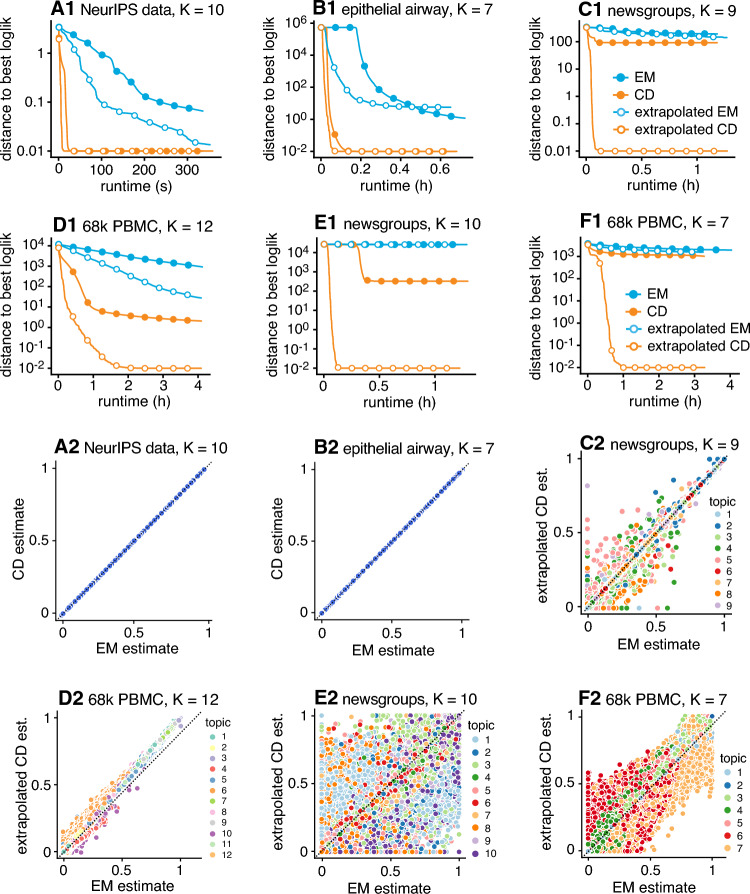
Selected results on fitting topic models using Poisson NMF algorithms. In A1–F1, multinomial topic model log-likelihoods are given relative to the best log-likelihood obtained among the four algorithms compared (EM and CD, with and without extrapolation). Log-likelihood differences less than 0.01 are shown as 0.01, and circles are drawn at intervals of 100 iterations. The 1,000 EM iterations performed during the initialization phase are not shown. Plots A2–F2 compare the final estimates of $\textbf{L}$ from each of A1–F1. See also Figures 5–12 in the Appendix for additional results obtained with different settings of *K*.

**Fig. 3:**
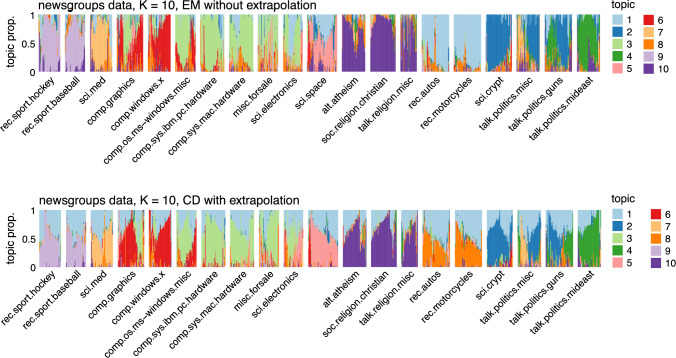
Estimates of $\textbf{L}$ from the newsgroups data with $K = 10$ obtained by running the EM updates without extrapolation (top) and the CD updates with extrapolation (bottom). The estimates of $\textbf{L}$ are visualized using Structure plots. The documents are arranged by newsgroup to show the correspondence between the newsgroups and the topics. Note that the ordering of the documents within each newsgroup is not exactly the same in the top and bottom plots. See E1 and E2 in Fig. 2 for related results.

**Fig. 4:**
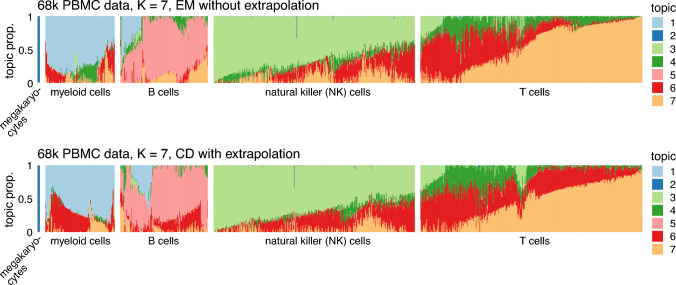
Estimates of $\textbf{L}$ from the 68k PBMC data with $K = 7$ obtained by running the EM updates without extrapolation (top) and the CD updates with extrapolation (bottom). The estimates of $\textbf{L}$ are visualized using Structure plots. To facilitate comparison, the cells were split into 5 groups based on the CD estimates of $\textbf{L}$; these groups roughly correspond to cell types (B cells, T cells, *etc*). The “T cells” group was downsampled to better visualize the other groups. Note that the ordering of the cells within each grouping is not exactly the same in the top and bottom plots. See F1 and F2 in Fig. 2 for related results

**Fig. 5:**
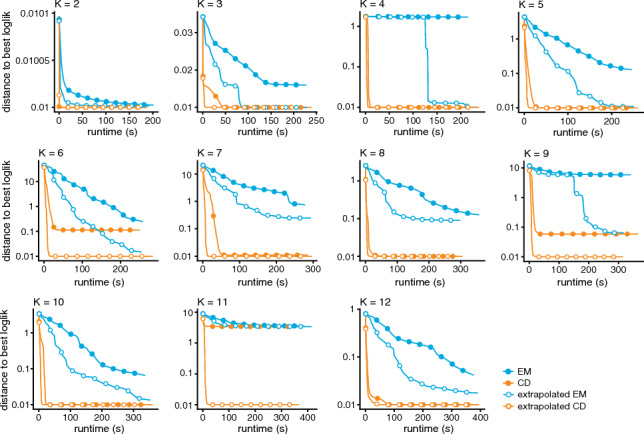
Improvement in model fit over time for the different Poisson NMF algorithms applied to the NeurIPS data. Multinomial topic model log-likelihoods are shown relative to the best log-likelihood recovered among the four algorithms compared (EM and CD, with and without extrapolation). Log-likelihood differences less than 0.01 are shown as 0.01. Circles are drawn at intervals of 100 iterations. Note that the 1,000 EM iterations performed during the initialization phase are not shown.

**Fig. 6:**
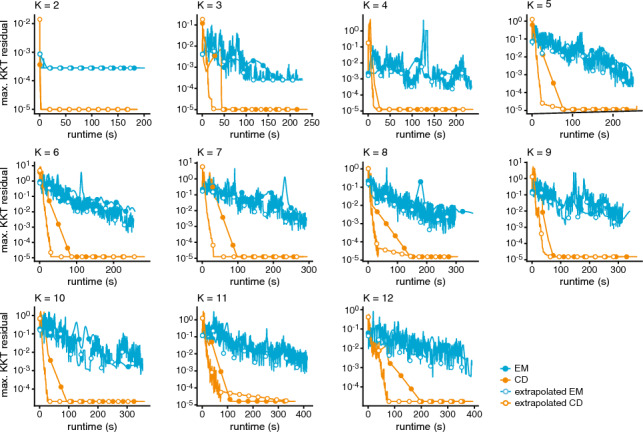
Evolution of the KKT residuals over time for the different Poisson NMF algorithms applied to the NeurIPS data. The KKT residuals should vanish near a local maximum of the Poisson NMF log-likelihood, so looking at the largest KKT residual can be used to assess how closely the algorithm recovers a stationary point (*i.e.*, an MLE). Note that the KKT residuals are not expected to decrease monotonically over time. Circles are drawn at intervals of 100 iterations.

**Fig. 7:**
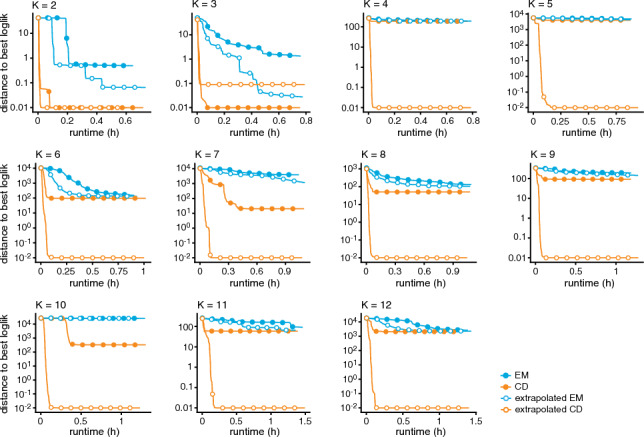
Improvement in model fit over time for the different Poisson NMF algorithms applied to the newsgroups data. See the Fig. 5 caption for more details.

**Fig. 8:**
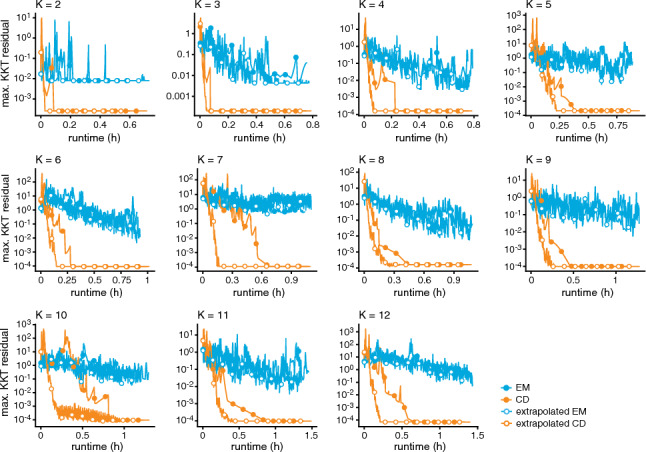
Evolution of the KKT residuals over time for the different Poisson NMF algorithms applied to the newsgroups data. See the Fig. 6 caption for more details.

**Fig. 9:**
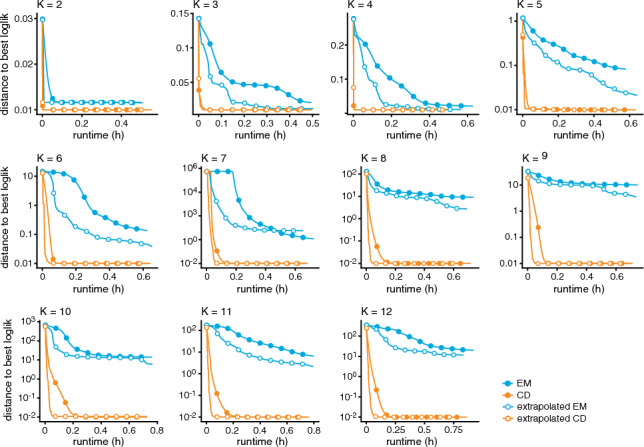
Improvement in model fit over time for the different Poisson NMF algorithms applied to the epithelial airway data. See the Fig. 5 caption for more details.

**Fig. 10:**
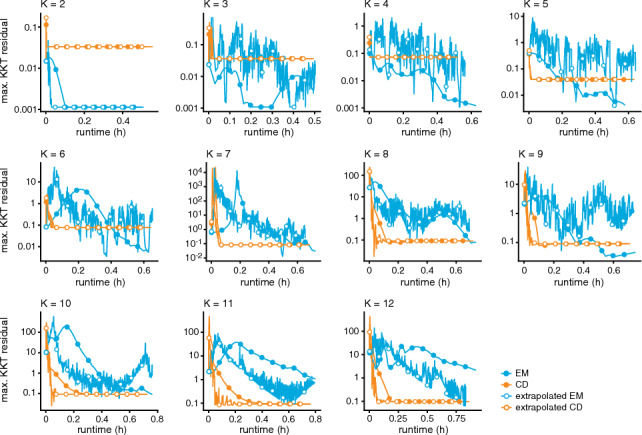
Evolution of the KKT residuals over time for the different Poisson NMF algorithms applied to the epithelial airway data. See the Fig. 6 caption for more details.

**Fig. 11:**
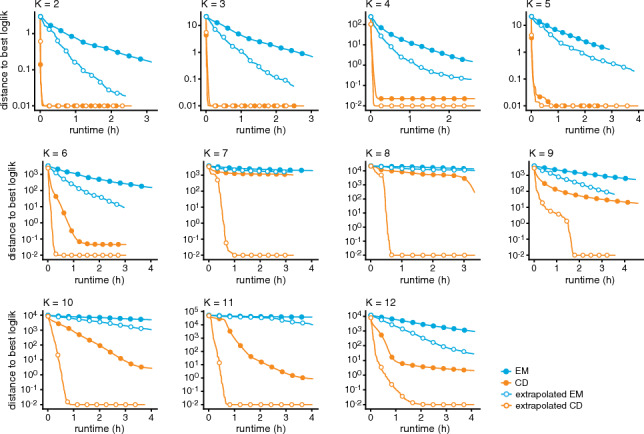
Improvement in model fit over time for the different Poisson NMF algorithms applied to the 68k PBMC data. See the Fig. 5 caption for more details.

**Fig. 12:**
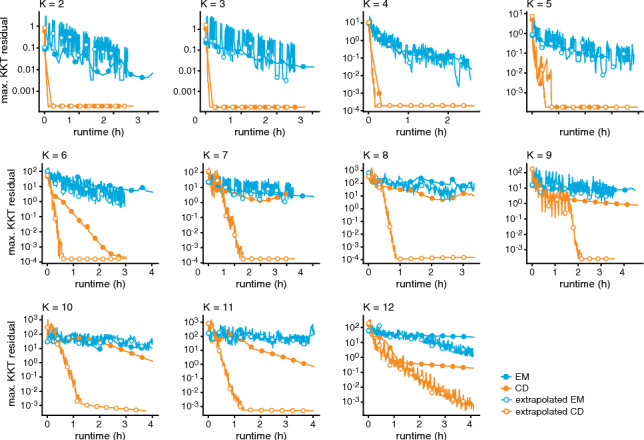
Evolution of the KKT residuals over time for the different Poisson NMF algorithms applied to the 68k PBMC data. See the Fig. 6 caption for more details.

Selected results of these comparisons are shown in Fig. 2, and more comprehensive results on all four data sets, with *K* ranging from 2 to 12, are given in the Appendix (Figures 5–12). In almost all cases, the extrapolated CD updates converged to an MLE at least as fast as the other algorithms, and often much faster, or produced the best fit within the allotted time. The extrapolation method generally helped convergence of CD, and sometimes helped EM. Also, the per-iteration running time per was very similar in all the algorithms. Beyond this, there was considerable variation in the algorithms’ performance among the different data sets and within each data set at different settings of *K*. To make sense of the diverse results, we distinguish three main patterns.

A1 and B1 in Fig. 2 illustrate the first pattern: EM quickly progressed to a good solution, and so any improvements over EM were small regardless of the algorithm used. Indeed, despite the small improvements in log-likelihood obtained by the CD estimates in A1 and B1, the final EM and CD estimates were nearly indistinguishable from each other (Fig. 2, A2 and B2).

C1 and D1 in Fig. 2 illustrate the second pattern: the initial 1,000 EM iterations were insufficient to recover estimates close to an MLE, and running additional updates sometimes substantially improved the fit. Among the four algorithms compared, the extrapolated CD updates again provided the greatest improvement in log-likelihood within the allotted time. And yet, despite the considerable improvements in log-likelihood, the final estimates did not change much (Fig. 2, C2 and D2). So while the CD updates can sometimes produce large gains in computational performance, these gains do not always have a meaningful impact on the topic modeling results.

E1 and F1 in Fig. 2 are examples of the third pattern: the extrapolated CD updates not only produced estimates with greatly improved log-likelihood, they also produced estimates that were *qualitatively very different* (Fig. 2, E2 and F2). In both this and the previous pattern, the EM updates progressed slowly toward a solution. But whereas this slow progress was benign in the previous examples, with little impact on the final result, in these examples the slow progress of EM was in an area of the likelihood that was very far away from an MLE. We also observed an example of this pattern earlier in the MCF-7 data set (Fig. 1). In brief, the slow convergence of the EM updates is sometimes benign, and sometimes not, but it is impossible to know in advance which it is without making these comparisons. Therefore, one way to avoid this problem is to use the CD updates, which are generally better at not getting stuck in areas of the likelihood far away from an MLE.

We also examined the topic model estimates in E and F to understand how the improved estimates can affect our understanding of the data. In the newsgroups data, topics 1 and 8 changed most between the EM and CD estimates (Fig. 3): in the EM estimates, the rec.auto and rec.motorcycle newsgroup discussions were largely captured by topic 1, a topic that was also shared by most other newsgroups; in the CD estimates, topic 8 distinguished rec.auto and rec.motorcycle from the other newsgroups, and topic 1 was present more evenly in all the newsgroups.

In the 68k PBMC data (Fig. 4), there were many differences between the EM and CD estimates, but the changes to topic 4 most affect our understanding of these data: in the EM estimates, the T cells shared topic 4 with a subset of myeloid cells—suggesting some sort of pathway or gene expression program common to myeloid and T cells—but in the improved CD estimates, this connection between myeloid and T cells mostly disappeared, and topic 4 was mostly distinct to T cells.

## Concluding remarks

In this paper, we suggested a simple strategy for fitting topic models by exploiting the equivalence of Poisson NMF and the multinomial topic model: first fit a Poisson NMF, then recover the corresponding topic model. To our knowledge, this equivalence, despite being informally recognized early on in the development of these methods, has not been previously exploited to fit topic models. The greatest improvements in optimization performance were achieved when the Poisson NMF was optimized using a simple co-ordinate descent (CD) algorithm. While the CD algorithm may be simple, consider that, due to the “sum-to-one” constraints, it is not obvious how to implement CD for the multinomial topic model.

For many statistical applications, point estimation such as maximum-likelihood estimation will suffice when the main aim is to learn a low-rank representation of the count data. (See also (Taddy 2012).) Further, focussing on point estimation simplifies numerical computation, allowing for simpler and more efficient algorithms that can be quickly applied to large data sets. We focussed on maximum-likelihood estimation, but the ideas and algorithms presented here also apply to MAP estimation with Dirichlet priors on $\textbf{F}$. Extending these ideas to improve variational inference algorithms for topic models (e.g., LDA) may also be of interest. Given the success of the CD approach, it may be fruitful to develop CD-based variational inference algorithms for LDA and Poisson NMF (Gopalan et al. 2015). That said, in many applications topic models are mainly used for dimension reduction—the goal being to learn compact representations of complex patterns—and in these applications maximum-likelihood or MAP estimation may suffice. Some topic modeling applications involve massive data sets that require an “online” approach (Hoffman et al. (2010); Sato (2001); Hoffman et al. (2013); Broderick et al. (2013)). Developing online versions of our algorithms is straightforward in principle, although online learning brings additional practical challenges, such as the choice of learning rate.

It is well known that EM can suffer from slow convergence; recent theoretical developments shed light on this slow convergence and the conditions under which it occurs (Kunstner et al. 2021; Dwivedi et al. 2020). And so it is perhaps not surprising that the EM variant of the Alternating Poisson Regression algorithm (Algorithm 1) was also very slow in some data sets. However, we distinguished between two types of slow convergence: benign slow convergence that occurs when the EM estimates are near an MLE; and slow convergence far away from an MLE, which can result in EM estimates that are very different from an MLE, and can affect how the topics are interpreted. Several methods have been developed specifically to accelerate EM (Zhou et al. (2011); Varadhan and Roland (2008); Henderson and Varadhan (2019)), and therefore it would have been natural to apply these methods here to improve the performance of the EM updates. We actually tried two more recent acceleration methods—DAAREM (Henderson and Varadhan 2019) and the quasi-Newton method of Zhou et al. (2011)—but in our tests (results not shown) we found that both methods provided little improvement over the unaccelerated EM. Taddy (2012) also used a quasi-Newton method to accelerate EM, but did not provide any results to show that this was beneficial. The only acceleration method that consistently improved performance was the extrapolation method of Ang and Gillis (2019).

## Data Availability

No datasets were generated or analysed during the current study.

## Appendix A Derivations and additional theory

### MAP estimation

#### Corollary 2

(Relationship between MAP estimates for Poisson NMF and the multinomial topic model) Let ${\hat{\textbf{H}}} \in \textbf{R}_{+}^{n \times K}, \hat{\textbf{W}} \in \textbf{R}_{+}^{m \times K}$ denote *maximum a posteriori* (MAP) estimates for the Poisson NMF model, in which elements of $\textbf{W}$ are assigned independent gamma priors, $w_{jk} \sim \textrm{Gamma}(a_{jk}, b_k)$, with $a_{jk} > 1$, $b_k > 0$, for $j = 1, \ldots , m$, $k = 1, \ldots , K$, and elements of $\textbf{H}$ are assigned an (improper) uniform prior,

A1 $$\begin{aligned} \hat{\textbf{H}}, \hat{\textbf{W}} \in \underset{\textbf{H},\,\textbf{W}}{\textrm{argmax}} \; p_{\textrm{PNMF}}(\textbf{X}\mid \textbf{H}, \textbf{W}) \, p_{\textrm{gam}}(\textbf{W}), \end{aligned}$$

in which

$$\begin{aligned} p_{\textrm{gam}}(\textbf{W})~{:}{=}~ \prod _{j=1}^m \prod _{k=1}^K \textrm{Gamma}(w_{jk}; a_{jk}, b_k), \end{aligned}$$

and where $\textrm{Gamma}(\theta ; \alpha , \beta ) \propto \theta ^{\alpha - 1} e^{-\beta \theta }$ denotes the probability density of the gamma distribution with shape $\alpha$ and rate (inverse scale) $\beta$. If $\hat{\textbf{L}}, \hat{\textbf{F}}$ are obtained by applying $\textsc {PNMF-\!to-\!MTM}$ to $\hat{\textbf{H}}, \hat{\textbf{W}}$, these are MAP estimates for the multinomial topic model with independent Dirichlet priors on the columns of $\textbf{F}$, $f_{1k}, \ldots , f_{mk} \sim \textrm{Dirichlet}(a_{1k}, \ldots , a_{mk})$ and a uniform prior on $\textbf{L}$,

A2 $$\begin{aligned} \hat{\textbf{L}},\hat{\textbf{F}} \in \underset{\textbf{L},\,\textbf{F}}{\textrm{argmax}} \, p_{\textrm{MTM}}(\textbf{X}\mid \textbf{L}, \textbf{F}) \, p_{\textrm{dir}}(\textbf{F}), \end{aligned}$$

in which

$$\begin{aligned} p_{\textrm{dir}}(\textbf{F})~{:}{=}~ \prod _{k=1}^K \textrm{Dirichlet}(f_{1k}, \ldots , f_{mk}; a_{1k}, \ldots , a_{mk}), \end{aligned}$$

and $\textrm{Dirichlet}(\theta _1, \ldots , \theta _d; \alpha _1, \ldots , \alpha _d) \propto \theta _1^{\alpha _1 - 1} \cdots \theta _d^{\alpha _d - 1}$ denotes the probability density of the Dirichlet distribution with parameters $\alpha _1, \ldots , \alpha _d$. Conversely, let $\hat{\textbf{L}} \in \textbf{R}_{\textrm{row}}^{n \times K}, \hat{\textbf{F}} \in \textbf{R}_{\textrm{col}}^{m \times K}$ denote multinomial topic model MAP estimates (A2), set $\hat{s}_i = t_i = \sum _{j=1}^m x_{ij}$, for $i = 1, \dots , n$, and set $\hat{u}_k = \sum _{j=1}^m (a_{jk}-1) / b_k$, for $k = 1, \dots , K$. Then if $\hat{\textbf{H}}, \hat{\textbf{W}}$ are obtained by applying $\textsc {MTM-\!to-\!PNMF}$ to $\hat{\textbf{L}}, \hat{\textbf{F}}, \hat{{\boldsymbol{s}}}, \hat{{\boldsymbol{u}}}$, these will be Poisson NMF MAP estimates (A1).

#### Proof

To prove this result, first note that minimizing the penalized objective for the multinomial topic model, $\phi ^{\star }(\textbf{X}; \textbf{L}, \textbf{F})$ (see eq. 17), corresponds to computing MAP estimates (A2). This penalized objective can be written in the form of the unpenalized objective, $\phi (\textbf{X}; \textbf{L}, \textbf{F})$, where $\tilde{\textbf{X}}$ and $\tilde{\textbf{L}}$ are matrices augmented with an additional *K* “pseudodocuments”,

A3 $$\begin{aligned} \tilde{\textbf{X}}~{:}{=}~ \left[ \begin{array}{c} \textbf{X} \\ \textbf{A}^T - 1 \end{array}\right] , \quad \tilde{\textbf{L}}~{:}{=}~ \left[ \begin{array}{c} \textbf{L} \\ \textbf{I}_K \end{array}\right] , \end{aligned}$$

and where $\textbf{A}$ is a $m \times K$ matrix with elements $a_{jk}$ and $\textbf{I}_K$ is the $K \times K$ identity matrix. In other words, each of the *K* pseudodocuments is attributed entirely to a single topic, and the Dirichlet prior parameters are treated as “pseudocounts”.

Similarly, the Poisson NMF penalized loss function $\ell ^{\star }(\textbf{X}; \textbf{H}, \textbf{W})$—in which minimizing this loss function corresponds to computing Poisson NMF MAP estimates (A1)—can be rewritten in the form of the unpenalized Poisson NMF objective function, $\ell (\textbf{X}; \textbf{H}, \textbf{W})$. To show this, we employ a change of variables that rescales the columns of $\textbf{H}$ and $\textbf{W}$, $\textbf{W}' \leftarrow \textbf{W} \textbf{U}^{-1}$, $\textbf{H}' \leftarrow \textbf{H} \textbf{U}$, where $\textbf{U}~{:}{=}~ \textrm{diag}({{\boldsymbol{u}}})$, and the columns of $\textbf{W}'$ are constrained to sum to one, $\sum _{j=1}^m w_{jk}' = 1$, $k = 1, \ldots , K$. With this change of variables, the penalized loss function can be rewritten as

$$\begin{aligned} \ell ^{\star }(\textbf{X}; \textbf{H}', \textbf{W}')&= \ell (\textbf{X}; \textbf{H}', \textbf{W}') \nonumber \\&\quad - \sum _{j=1}^m \sum _{k=1}^K (a_{jk} - 1) \log (u_k w_{jk}') \nonumber \\&\quad + \sum _{k=1}^K b_k u_k. \end{aligned}$$

With this objective, one can independently solve for each $u_k$, with analytic solution $\hat{u}_k = \sum _{j=1}^m (a_{jk} - 1)/b_k$ that does not depend on the other parameters. Therefore, we focus on the part of the loss function that depends on $\textbf{H}', \textbf{W}'$, that is,

$$\begin{aligned} \ell ^{\star }(\textbf{X}; \textbf{H}', \textbf{W}')&= \ell (\textbf{X}; \textbf{H}', \textbf{W}') \nonumber \\&\quad - \sum _{j=1}^m \sum _{k=1}^K (a_{jk} - 1) \log w_{jk}' + \text{ const }, \end{aligned}$$

where the “const” is a placeholder for terms that do not depend on $\textbf{H}'$ or $\textbf{W}'$. This can written as $\ell ^{\star }(\textbf{X}; \textbf{H}', \textbf{W}') = \ell (\tilde{\textbf{X}}; \tilde{\textbf{H}}'; \textbf{W}') + \text{ const }$, where $\tilde{\textbf{X}}$ is the data matrix augmented with pseudocounts (A3), and $$\tilde{\textbf{H}}'~{:}{=}~ \left[ \begin{array}{c} \textbf{H}' \\ \textbf{I}_K \end{array}\right] $$ is the matrix $\textbf{H}'$ augmented with pseudodocuments. Finally, we revert back to the original parameterization, $\tilde{\textbf{H}} \leftarrow \tilde{\textbf{H}}' \hat{\textbf{U}}^{-1}, \quad \textbf{W} \leftarrow \textbf{W}' \hat{\textbf{U}}$, where $\hat{\textbf{U}}~{:}{=}~ \textrm{diag}(\hat{{\boldsymbol{u}}})$.

To summarize, this shows that MAP estimation (A1, A2) can be reduced to MLE estimation (13, 14) when $\textbf{X}$ is replaced with $\tilde{\textbf{X}}$, and $\textbf{H}$ is replaced with $\tilde{\textbf{H}}$; that is,

A4 $$\begin{aligned}&\textrm{argmax}_{\textbf{H},\,\textbf{W}} \; p_{\textrm{PNMF}}(\textbf{X}\mid \textbf{H}, \textbf{W}) \, p_{\textrm{gam}}(\textbf{W}) \nonumber \\&\quad = \textrm{argmax}_{\textbf{H}, \, \textbf{W}} \; p_{\textrm{PNMF}}(\tilde{\textbf{X}} \mid \tilde{\textbf{H}}, \textbf{W}) \end{aligned}$$

A5 $$\begin{aligned}&\textrm{argmax}_{\textbf{L},\,\textbf{F}} \; p_{\textrm{MTM}}(\textbf{X}\mid \textbf{L}, \textbf{F}) \, p_{\textrm{dir}}(\textbf{F}) \nonumber \\&\quad = \textrm{argmax}_{\textbf{L}, \, \textbf{F}} \; p_{\textrm{MTM}}(\tilde{\textbf{X}} \mid \tilde{\textbf{L}}, \textbf{F}). \end{aligned}$$

Therefore, we can apply Corollary 1 to prove Corollary 2. $\square$

### EM algorithms

#### EM for the additive Poisson regression model

Here we rederive the basic EM algorithm (Lange and Carson (1984); Shepp and Vardi (1982); Vardi et al. (1985)) for fitting the additive Poisson regression model (23). To do so, we first introduce a data-augmented version of the Poisson regression model:

A6 $$\begin{aligned} \begin{aligned} z_{ik}&\sim \textrm{Pois}(a_{ik} b_k) \\ y_i&= \textstyle \sum _{k=1}^K z_{ik}. \end{aligned} \end{aligned}$$

Under this data-augmented model, the expected complete log-likelihood is

A7 $$\begin{aligned} E[\log p({{\boldsymbol{y}}}, {{\boldsymbol{z}}} \mid \textbf{A}, {{\boldsymbol{b}}})]&= \sum _{i=1}^n \sum _{k=1}^K \bar{z}_{ik} \log (a_{ik} b_k) \nonumber \\&\quad - \sum _{i=1}^n \sum _{k=1}^K a_{ik} b_k + \text{ const }, \end{aligned}$$

where “const” includes additional terms in the likelihood that do not depend on ${{\boldsymbol{b}}}$, and $\bar{z}_{ik} = E[z_{ik}]$ is the expected value of $z_{ik}$ with respect to the posterior $p({{\boldsymbol{z}}} \mid \textbf{A}, {{\boldsymbol{b}}}, {{\boldsymbol{y}}})$.

Using this data-augmented model, the M step (26) is derived by taking the partial derivative of (A7) with respect to $b_k$, and solving for $b_k$. The E step (26) involves computing posterior expectations at the current ${{\boldsymbol{b}}} = (b_1, \ldots , b_K)$. The posterior distribution of $z_i = (z_{i1}, \ldots , z_{iK})$ is multinomial with $y_i$ trials and multinomial probabilities $p_{ik} \propto a_{ik} b_k$. Therefore, the posterior expected value of $z_{ik}$ is

A8 $$\begin{aligned} \bar{z}_{ik} = y_i p_{ik} = y_i a_{ik} b_k / \mu _i. \end{aligned}$$

The EM algorithm iterates the E and M steps until some stopping criterion is met. Alternatively, the E and M steps can be combined, yielding the update

A9 $$\begin{aligned} b_k^\textrm{new} \leftarrow b_k \times \frac{\sum _{i=1}^n a_{ik} y_i/\mu _i}{\sum _{i=1}^n a_{ik}}. \end{aligned}$$

#### Alternative EM algorithm for additive Poisson regression

The additive Poisson regression model (23) is equivalent to a *multinomial mixture model* by a simple reparameterization. The multinomial mixture model is

A10 $$\begin{aligned} \begin{array}{r@{\;}c@{\;}l} y_1, \ldots , y_n & \sim & \textrm{Multin}(t, {{\boldsymbol{\pi }}}), \\ \pi _i & =& \sum _{k=1}^K a_{ik}' b_k', \end{array} \end{aligned}$$

in which $\textbf{A}' \in \textbf{R}_{+}^{n \times K}$, ${{\boldsymbol{y}}} = (y_1, \ldots , y_n) \in \textbf{R}_{+}^n$, ${{\boldsymbol{\pi }}} = (\pi _1, \ldots , \pi _n)$ and $t = \sum _{i=1}^n y_i$. To ensure that the $\pi _i$’s are probabilities, we require $b_k' \ge 0$, $a_{ik}' \ge 0$, $\sum _{k=1}^K b_k' = 1$, $\sum _{i=1}^n a_{ik}' = 1$. The multinomial mixture model is a reparameterization of the Poisson regression model (23) that preserves the likelihood; that is,

A11 $$\begin{aligned} \prod _{i=1}^n \textrm{Pois}(y_i; \mu _i) = \textrm{Multin}({{\boldsymbol{y}}}; t, {{\boldsymbol{\pi }}}) \, \textrm{Pois}(t; s), \end{aligned}$$

in which the right-hand side quantities $\textbf{A}', {{\boldsymbol{b}}}', s$ are recovered from the left-hand side quantities $\textbf{A}, {{\boldsymbol{b}}}$ as follows:

A12 $$\begin{aligned} \begin{aligned} u_k&\leftarrow \textstyle \sum _{i=1}^n a_{ik} \\ s&\leftarrow \textstyle \sum _{k=1}^K b_k u_k \\ a_{ik}'&\leftarrow a_{ik} / u_k \\ b_k'&\leftarrow b_k u_k / s. \end{aligned} \end{aligned}$$

The EM algorithm for fitting the multinomial mixture model consists of iterating the following E and M steps:

A13 $$\begin{aligned} p_{ik}&= \frac{a_{ik}' b_k'}{\sum _{j=1}^K a_{ij}' b_j'} \end{aligned}$$

A14 $$\begin{aligned} b_k'&= \frac{1}{t} \sum _{i=1}^n y_i p_{ik}. \end{aligned}$$

Once the multinomial mixture model parameters $b_1', \ldots , b_K'$ have been updated by performing one or more EM updates, the Poisson regression model parameters $b_1, \ldots , b_K$ are recovered as $b_k = t b_k'/u_k$, using the MLE of *s*, $s = t$.

#### Multiplicative updates for Poisson NMF

Having derived EM for the additive Poisson regression model in the previous section, we can use this result to re-derive the multiplicative updates for Poisson NMF (Lee and Seung 2001) by making substitutions $\textbf{A} \leftarrow \textbf{W}$, ${{\boldsymbol{y}}} \leftarrow {{\boldsymbol{x}}}_i$, ${{\boldsymbol{b}}} \leftarrow {{\boldsymbol{h}}}_i$ in (A9), where ${{\boldsymbol{h}}}_i$ denotes a row of $\textbf{H}$ and ${{\boldsymbol{x}}}_i$ denotes a row of $\textbf{X}$, the update becomes the Poisson NMF multiplicative update for row *i* of $\textbf{H}$; similarly, making substitutions $\textbf{A} \leftarrow \textbf{H}$, ${{\boldsymbol{y}}} \leftarrow {{\boldsymbol{x}}}_j$, ${{\boldsymbol{b}}} \leftarrow {{\boldsymbol{w}}}_j$ in (A9), where ${{\boldsymbol{w}}}_j$ denotes a row of $\textbf{W}$ and ${{\boldsymbol{x}}}_j$ denotes a column of $\textbf{X}$, the update becomes the Poisson NMF multiplicative update for row *j* of $\textbf{W}$.

#### EM for the multinomial topic model

Here we derive EM for the multinomial topic model (Hofmann 2001), and connect EM for the multinomial topic model and the multiplicative updates for Poisson NMF. The EM algorithm for the multinomial topic model is based on a data-augmented version of the topic model (Blei et al. 2003),

A15 $$\begin{aligned} \begin{aligned} p(z_{it} = k \mid \textbf{L})&= l_{ik} \\ p(d_{it} = j \mid \textbf{F}, z_{it} = k)&= f_{jk}, \end{aligned} \end{aligned}$$

where $d_{it} \in \{1, \ldots , K\}$, and the data are $d_{it} \in \{1, \ldots , m\}$, $t = 1, \ldots , n_i$, in which $n_i$ is the size of document *i*. Summing over the topic assignments $z_{ij}$ recovers the multinomial topic model (6), in which the word counts are recovered as $x_{ij} = \sum _{t = 1}^{n_i} \delta _j(d_{it})$.

The E step consists of computing the posterior expected values for the latent topic assignments $z_{ij}$,

A16 $$\begin{aligned} p_{ijk}&~{:}{=}~ p(z_{ij} = k \mid {\textbf{X}}, \textbf{L}, \textbf{F}) = l_{ik} f_{jk}/\pi _{ij}. \end{aligned}$$

The M step for the topic proportions $l_{ik}$ and word frequencies $f_{jk}$ is

A17 $$\begin{aligned} l_{ik}&= \sum _{j=1}^m x_{ij} p_{ijk} / n_i \end{aligned}$$

A18 $$\begin{aligned} f_{jk}&\propto \sum _{i=1}^n x_{ij} p_{ijk}. \end{aligned}$$

Combining the E and M steps, we obtain the following updates:

A19 $$\begin{aligned} l_{ik}^\textrm{new}&\leftarrow \frac{l_{ik}}{t_i} \sum _{j=1}^m x_{ij} f_{jk}/\pi _{ij} \end{aligned}$$

A20 $$\begin{aligned} f_{jk}^\textrm{new}&\leftarrow \frac{f_{jk}}{\xi _k} \sum _{i=1}^n x_{ij} l_{ik} / \pi _{ij}. \end{aligned}$$

Here, $\xi _k > 0$ is a normalizing factor that ensures that $\sum _{j=1}^m f_{jk}^\textrm{new} = 1$. To connect to Poisson NMF, these updates can also be derived by applying $\textsc {PNMF-\!to-\!MTM}$ and afterward its inverse to the Poisson NMF multiplicative updates (27, 28).

### KKT conditions

The first-order KKT conditions for the Poisson NMF optimization problem (2) are

A21 $$\begin{aligned} \nabla _\textbf{H} \mathcal {L}(\textbf{X}; \textbf{H}, \textbf{W}, \mathbf{\Gamma }, \mathbf{\Omega })&= {{\boldsymbol{0}}} \end{aligned}$$

A22 $$\begin{aligned} \nabla _\textbf{W} \mathcal {L}(\textbf{X}; \textbf{H}, \textbf{W}, \mathbf{\Gamma }, \mathbf{\Omega })&= {{\boldsymbol{0}}} \end{aligned}$$

A23 $$\begin{aligned} \mathbf{\Gamma } \odot \textbf{H}&= {{\boldsymbol{0}}} \end{aligned}$$

A24 $$\begin{aligned} \mathbf{\Omega } \odot \textbf{W}&= {{\boldsymbol{0}}} \end{aligned}$$

in which $\mathcal {L}(\textbf{X}; \textbf{H}, \textbf{W}, \mathbf{\Gamma }, \mathbf{\Omega })$ denotes the Lagrangian function,

$$\begin{aligned} \mathcal {L}(\textbf{X}; \textbf{H}, \textbf{W}, \mathbf{\Gamma }, \mathbf{\Omega }) ~{:}{=}~\;&\ell (\textbf{X}; \textbf{H}, \textbf{W}) - \Vert \mathbf{\Gamma } \odot \textbf{H} \Vert _{1,1} \nonumber \\ &- \Vert \mathbf{\Omega } \odot \textbf{W} \Vert _{1,1}. \end{aligned}$$

To define the Lagrangian function, we have introduced two matrices of Lagrange multipliers, $\mathbf{\Gamma } \in \textbf{R}_{+}^{n \times K}$ and $\mathbf{\Omega } \in \textbf{R}_{+}^{m \times K}$, associated with the non-negativity constraints $\textbf{H} \ge {{\boldsymbol{0}}}$ and $\textbf{W} \ge {{\boldsymbol{0}}}$. Combining (A21) and (A22), we obtain

A25 $$\begin{aligned} \begin{aligned} \mathbf{\Omega }&= (1 - \textbf{U})^T\textbf{H} \\ \mathbf{\Gamma }&= (1 - \textbf{U})\textbf{W}, \end{aligned} \end{aligned}$$

in which $\textbf{U}$ is an $n \times m$ matrix with entries $u_{ij} = x_{ij} / \lambda _{ij}$. See also Dhillon and Sra (2005) and Févotte and Idier (2011) for generalizations of these KKT conditions.

## Appendix B Algorithm implementation and enhancements

### Extrapolated updates

To accelerate convergence of the EM and CD updates, we used the extrapolation method of Ang and Gillis (2019). In brief, at iteration *t*, the extrapolated update is

B26 $$\begin{aligned} \begin{aligned} \textbf{H}^{\textrm{ext}}&\leftarrow P_{+}[\textbf{H}^{\textrm{new}} + \beta ^{(t)}(\textbf{H}^{\textrm{new}} - \textbf{H}^{(t-1)})] \\ \textbf{W}^{\textrm{ext}}&\leftarrow P_{+}[\textbf{W}^{\textrm{new}} + \beta ^{(t)}(\textbf{W}^{\textrm{new}} - \textbf{W}^{(t-1)})], \end{aligned} \end{aligned}$$

where $\textbf{H}^{\textrm{new}}$ is a new estimate obtained by solving $\textsc {Fit-\!Pois-\!Reg}(\textbf{W}^{(t-1)}, {{\boldsymbol{x}}}_i)$ for each $i = 1, \ldots , n$, $\textbf{W}^{\textrm{new}}$ is a new estimate obtained by $\textsc {Fit-\!Pois-\!Reg}(\textbf{H}^{\textrm{ext}}, {{\boldsymbol{x}}}_j)$ for each $j = 1, \ldots , m$, $P_{+}(\textbf{A})$ is the projection of matrix $\textbf{A}$ onto the non-negative orthant, $P_{+}(\textbf{A})_{ij}~{:}{=}~ \max \{0,(\textbf{A})_{ij}\}$, and $\beta ^{(t)} \in [0,1]$ is a parameter that interpolates between the new estimate and the estimate from the previous iteration. (Note that setting $\beta ^{(t)} = 0$ recovers the update with no extrapolation.) Although Ang and Gillis (2019) developed the extrapolation method for Frobenius-norm NMF, our initial trials showed that it also worked well for Poisson NMF. It also worked better than the other acceleration schemes we tried, the damped Anderson (DAAREM) method of Henderson and Varadhan (2019) and the quasi-Newton acceleration method of Zhou et al. (2011).

### Other enhancements and implementation details

Here we detail other steps taken to speed up computation and improve numerical stability of the Poisson NMF optimization algorithms.

**Computations with sparse data.** Topic modeling data sets often have very high levels of sparsity; that is, most of the counts $x_{ij}$ are zero. We therefore used sparse matrix computation techniques to reduce computational effort for sparse data sets. To illustrate the importance of sparse computations, consider computing the Poisson NMF loss function $\ell (\textbf{X}; \textbf{H}, \textbf{W})$ when $\textbf{X}$ is sparse. Once the $\Vert \textbf{H} \textbf{W}^T\Vert _{1,1}$ term is computed, which requires $O((n + m)K)$ operations, computing $\phi (\textbf{X}; \textbf{H}, \textbf{W})$ requires and additional *O*(*NK*) operations, where *N* is the number of nonzeros in $\textbf{X}$, because terms in the sum corresponding to $x_{ij} = 0$ can be ignored. Therefore, for sparse $\textbf{X}$ the time complexity of computing $\ell (\textbf{X}; \textbf{H}, \textbf{W})$ is $O((N + n + m)K)$. Without considering sparsity, the time complexity is *O*(*nmK*), which is much greater than $O((N + n + m)K)$ when $n \times m \gg N$. Similar logic applies to other computations such as gradients and EM (multiplicative) updates.

**Incomplete optimization of subproblems.** In practice, accurately solving each subproblem $\textsc {Fit-\!Pois-\!Reg}(\textbf{L}, {{\boldsymbol{x}}}_j)$ and $\textsc {Fit-\!Pois-\!Reg}(\textbf{F}, {{\boldsymbol{x}}}_i)$ may not be necessary, particularly in initial stages when $\textbf{H}$ and $\textbf{W}$ change a lot from one iteration to the next. Incompletely solving the subproblems has been shown to work well for Frobenius-norm NMF (Gillis and Glineur 2012; Hsieh and Dhillon 2011; Kim et al. 2014). Therefore, instead of running the EM or CD algorithm to convergence, we stopped the optimization early when the number of iterations exceeded some limit. We found that performing at most 4 EM or CD updates worked well in practice when initialized to the estimate from the previous (outer-loop) iteration. We write this incomplete optimization as $\textsc {Fit-\!Pois-\!Reg}(\textbf{A}, \textbf{y}, \textbf{b}_0)$, where ${{\boldsymbol{b}}}_0$ makes explicit the dependence on an initial estimate. Therefore, when the subproblems are solved incompletely, $\textsc {Fit-\!Pois-\!Reg}(\textbf{W}^{(t-1)}, {{\boldsymbol{x}}}_i)$ is replaced with $\textsc {Fit-\!Pois-\!Reg}(\textbf{W}^{(t-1)}, {{\boldsymbol{x}}}_i, {{\boldsymbol{h}}}_i^{(t-1)})$ in Algorithm 1, and $\textsc {Fit-\!Pois-\!Reg}(\textbf{H}^{(t)}, {{\boldsymbol{x}}}_j)$ is replaced with $\textsc {Fit-\!Pois-\!Reg}(\textbf{H}^{(t)}, {{\boldsymbol{x}}}_j, {{\boldsymbol{w}}}_j^{(t-1)})$.

**Parallel computations.** We used Intel Threading Building Blocks (TBB) multithreading (Contreras and Martonosi 2008) to solve $\textsc {Fit-\!Pois-\!Reg}(\textbf{W}, {{\boldsymbol{x}}}_i)$, $i = 1, \ldots , n$, and $\textsc {Fit-\!Pois-\!Reg}(\textbf{H}, {{\boldsymbol{x}}}_j)$, $j = 1, \ldots , m$, in parallel.

**Refinement of CD updates.** Since the CD updates were performed without a line search, we found that the CD updates sometimes failed to improve the objective when the iterate was far away from a solution. Therefore, to reduce the failure rate of the CD updates, we performed a single EM update prior to running each CD update.

**Improved convergence guarantees.** Following Gillis and Glineur (2012), any parameter estimates that fell below $10^{-15}$ were set to this value.

**Rescaled updates.** To improve numerical stability of the updates, as others have done (e.g., Lee and Seung 1999)), we rescaled $\textbf{W}$ and $\textbf{H}$ after each full update. Specifically, we rescaled the matrices so that the column means of $\textbf{W}$ were equal to the column means of $\textbf{H}$. Note that the Poisson rates $\lambda _{ij} = (\textbf{H} \textbf{W}^T)_{ij}$ and the Poisson NMF loss function $\ell (\textbf{X}; \textbf{H}, \textbf{W})$ are invariant to this rescaling.

**Assessing convergence.** We used two measures to assess convergence of the iterates: (1) the change in the loss function $\ell (\textbf{X}; \textbf{H}, \textbf{W})$, which is the same as the change in the Poisson NMF log-likelihood; and (2) the maximum residual of the KKT conditions.

## Appendix C Details of the numerical experiments

### Data sets

The NeurIPS (Globerson et al. 2007) and newsgroups (Rennie 2007) data sets are word counts extracted from, respectively, 1988–2003 NeurIPS (formerly NIPS) papers and posts to 20 different newsgroups. The data sets were retrieved from http://ai.stanford.edu/~gal/data.html and http://qwone.com/~jason/20Newsgroups. Documents with fewer than 2 nonzero word counts were removed.

The MCF-7 data are RNA-sequencing read counts from human MCF-7 cells (Sanford et al. 2020). These data were downloaded from the Gene Expression Omnibus (GEO) website, accession GSE152749.

The epithelial airway and 68k PBMC data sets are UMI (unique molecular identifier) counts from single-cell RNA sequencing experiments in trachea epithelial cells in C57BL/6 mice (Montoro et al. 2018) and in “unsorted” human peripheral blood mononuclear cells (PBMCs) [Fresh 68k PBMC Donor A] (Zheng et al. 2017). The epithelial airway data were downloaded from GEO, accession GSE103354. Specifically, we downloaded file GSE103354_Trachea_droplet_UMIcounts.txt.gz. Genes that were not expressed in any of the cells were removed. For the 68k PBMC data, we downloaded the “Gene/cell matrix (filtered)” tar.gz file for the Fresh 68k PBMCs (Donor A) data set from the 10x Genomics website (https://www.10xgenomics.com/datasets). Genes that were not expressed in any of the cells were removed.

All data sets except the MCF-7 data set were stored as sparse $n \times m$ count matrices $\textbf{X}$, where *n* is the number of documents or cells, and *m* is the number of words or genes. The MCF-7 data were not sparse, so they were stored as a dense matrix. The data processing scripts are provided in the Zenodo repository (Carbonetto et al. 2024).

### Computing environment

All computations on real data sets were run in R 3.5.1 (R Core Team 2018), linked to the OpenBLAS 0.2.19 optimized numerical libraries, on Linux machines (Scientific Linux 7.4) with Intel Xeon E5-2680v4 (“Broadwell”) processors. For running the Poisson NMF optimization algorithms, which included some multithreaded computations, 8 CPUs and as much as 16 GB of memory were used.

### Source code and software

The methods described in this paper were implemented in the fastTopics R package. The main numerical results (other than the in-depth illustration with the MCF-7 data set) were generated using version 0.5-24 of the R package. The core optimization algorithms were developed in C++ and interfaced to R using Rcpp (Eddelbuettel and François 2011). The CD updates were adapted from the C++ code included with the NNLM R package, version 0.4-3 (Lin and Boutros 2020). The Zenodo repository (Carbonetto et al. 2024) (see also https://github.com/stephenslab/fastTopics-experiments/) contains the code implementing the numerical experiments, and a workflowr website (Blischak et al. 2019) for browsing the results.

## Appendix D Additional figures

Figures 5–12 contain additional results from the numerical experiments (See Figs. 7, 8, 9, 10, 11).

## Footnotes

1 Recent papers have also studied the problem of identifying “anchor words,” which are words that appear in exactly one topic. In this setting, there is also a close relationship between the algorithms for identifying anchor words and the algorithms for identifying “separable” non-negative factors Arora et al. (2012, 2013); Donoho and Stodden (2003); Gillis (2021); Gillis and Vavasis (2015).

2 In descriptions of NMF, it is more common to represent data vectors (e.g., documents) as *columns* of $\textbf{X}$ (e.g., (Gillis 2021; Kim et al. 2014; Lee and Seung 2001)), in which case one would write $\textbf{X} \approx \textbf{W} \textbf{H}^T$. Here, we represent documents as *rows* of $\textbf{X}$, following Hofmann (1999); Taddy (2012). Due to the symmetry of Poisson NMF (1), it makes no difference if we fit $\textbf{X} \approx \textbf{H} \textbf{W}^T$ or $\textbf{X}^T \approx \textbf{H} \textbf{W}^T$. It only matters when connecting Poisson NMF to the topic model.

3 The notation $\hat{\theta } \in \textrm{argmax}_{\theta } \, f(\theta )$ means $f(\hat{\theta }) \ge f(\theta )$ for all $\theta$, and accounts for the fact that an MLE may not be unique due to non-identifiability.
